# Evaluating the Accumulation of Grain Mercury in Engineered Rice Lines Containing *merA* and *merB* Genes Under an Organic Mercury-Enriched Condition

**DOI:** 10.3390/plants14010066

**Published:** 2024-12-28

**Authors:** Deyao Meng, Zhaoze Song, Jinyuan Liu, Liyang Ji, Chen Chen, Changhu Wang

**Affiliations:** 1 State Key Laboratory of Plant Diversity and Specialty Crops, South China Botanical Garden, Chinese Academy of Sciences, Guangzhou 510650, China; 2 University of the Chinese Academy of Sciences, Beijing 100049, China; 3 Key Laboratory of South China Agricultural Plant Molecular Analysis and Genetic Improvement & Guangdong Provincial Key Laboratory of Applied Botany, South China Botanical Garden, Chinese Academy of Sciences, Guangzhou 510650, China

**Keywords:** low mercury accumulation, *merA*, *merB*, organic mercury, *Oryza sativa* L.

## Abstract

Rice is a critical crop for human sustenance worldwide. Food security has increasingly attracted public concerns, particularly due to heavy metal pollution, which adversely impacts crop yield and quality, with cadmium and mercury being the primary culprits. Excessive soil mercury not only hampers rice’s growth and development but also leads to a substantial accumulation in grains, posing a significant threat to human health. To mitigate the issue, low-mercury germplasms in rice were developed by expressing bacterial *merA* and *merB* genes, which convert mercury to less toxic forms from its most hazardous organic form: methylmercury. While previous evaluations of transgenic lines were typically conducted in environments mimicking inorganic mercury enrichment, studies on their performance in organic mercury-rich conditions, such as year-round rice planting paddies, remain limited. In this study, *merA* and *merB* transgenic rice lines were cultivated in organic mercury-contaminated soil to evaluate their grain mercury accumulation. Results showed a reduction in total grain mercury contents across three transgenic lines. Notably, one combined *merA* and *merB* line exhibited decreased organic mercury accumulation, and a reduction in total mercury levels in its grains, highlighting its breeding potential as a low-mercury rice germplasm for breeding programs.

## 1. Introduction

Rice (*Oryza sativa* L.) is the staple food for half of the world population and grown mainly in Asia, southern Europe, and parts of tropical America and Africa [1], but it is threatened by increasing heavy metal pollution. Since soil microorganisms cannot decompose metals, metals can only be transferred from one chemical state to another and then persist in the environment for many years [2]. In 2014, the Ministry of Environmental Protection P.R.C. (MEP) and the Ministry of Land and Resources P.R.C. (MLR) released a report on the status of soil pollution in China, in which 19% of arable land is affected by inorganic and/or organic pollutants. The top inorganic pollutants are cadmium (Cd) and mercury (Hg), followed by arsenic (As), copper (Cu), lead (Pb), and zinc (Zn) [3].

Mercury, one of the most prevalent heavy metals in nature, poses significant risks to human health, particularly to the brain. It can cause neurological dysfunction, leading symptoms such as weakness, movement disorders, anorexia gastrointestinal issues, heart failure, and even death. While the global mercury content in soils ranges from 0.58 to 1.8 mg/kg, the average is approximately 1.1 mg/kg [4]. However, due to environmental factors, certain regions naturally experience mercury enrichment. In nature, mercury exists in three forms: elemental mercury, inorganic mercury (IHg), and organic mercury. These forms undergo physical and chemical transformations in the biogeochemical cycle [5]. Within the biosphere, mercury circulates between the atmosphere and the Earth surface. Elemental mercury is widespread in water and readily volatilizes from soil. Compared to elemental mercury, IHg and organic mercury are significantly more toxic, with organic mercury, particularly methylmercury (MeHg), being among the most hazardous. MeHg is an organic compound that easily combines with anions, such as Cl^−^, OH^−^, and NO₃^−^. Due to its potent neurotoxicity and resistance to degradation, MeHg accumulates in the food chain, posing severe threats to human beings and the environment [6]. Studies indicate that MeHg has a half-life that is approximately 50 days as a lipophilic substance. Daily excretion accounts for only 1.4% of the daily intake, leading to continuous accumulation over time. This persistent accumulation eventually becomes life-threatening [7].

Mercury’s harmful effects are not limited to humans; it also damages plant respiratory and photosynthetic systems and disrupts nutrient uptake, ultimately leading to yield loss [8]. To make matters worse, there is currently a lack of data on the metabolic conversion of mercury in plants. As a result, the mercury absorbed by plants retains its speciation from the environment and is substantially transmitted, accumulated, and amplified through the food chain [9].

Methylmercury in soil is more easily absorbed by organisms than inorganic mercury, and it is easily transported to above-ground tissues. As a result, rice roots accumulate less methylmercury, with most of the absorbed MeHg being transferred to the grains from the leaves and stems [10,11,12]. Transgenic plants with enhanced mercury resistance can be developed by incorporating bacteria mercury-resistant genes, such as a regulatory gene (*merR*) and functional genes (*merT*, *merP*, *merD*, *merF*, *merC*, *merA* and *merB*) [5]. Of them, the *merA* gene encodes a mercury reductase that reduces Hg(II) to Hg(0) by using NAD(P)H as a reducing agent [13]. Studies have shown that merA confers mercury resistance to plants. For instance, transgenic Arabidopsis expressing the merA gene successfully germinated and grew on a medium containing inorganic mercury Hg(II), whereas wild-type seeds could not. This demonstrated that heterologous expression of *merA* enhanced mercury resistance in Arabidopsis [14]. When the *merA* transgenic rice was grown on the medium containing Hg(II), it exhibited significantly higher mercury resistance compared to that of wild type plants, with reduced mercury accumulation in their tissues. These findings support the potential of *merA* in breeding rice germplasms with low mercury accumulation [15]. The *merB* gene encodes an organic mercury lyase that catalyzes the cleavage of carbon–mercury bonds, breaking down organic mercury into ionic mercury and reduced hydrocarbons [5]. Studies on transgenic Arabidopsis expressing *merB* revealed that, even at relatively low expression levels, these plants exhibited resistance to organic mercury stress.

To reduce mercury levels in plants, the *merB* and *merA* genes are combined to sequentially detoxify different forms of mercury. Studies have shown that the combined action of these two genes in Arabidopsis can convert organic mercury to elemental mercury, which is subsequently volatilized into the air [16]. A study showed that only plants with both *merA* and *merB* genes could grow on a medium containing a high concentration of organic mercury PMA (phenylmercuric acetate). In contrast, plants transformed with either *merA* or *merB* alone showed limited resistance. Specifically, *merB*-transformed plants could germinate under these conditions, but gradually developed chlorosis and eventually died. On the other hand, *merA*-transformed plants only survived on mercury-free medium, highlighting the significantly higher toxicity of organic mercury, compared to that of inorganic mercury. A similar outcome was observed in transgenic tobacco study, where plants expressing both genes exhibited greater resistance to high mercury stress than wild-type plants, regardless of whether they were exposed to inorganic or organic mercury [17]. These findings underscore the potential of *merB* and *merA* gene combination in addressing mercury pollution in plants across diverse scenarios.

Although most studies have suggested that the main source of mercury in the human diet is fish and fish-related products containing MeHg [18], MeHg was found to be abundant in rice grains ten years ago in some areas and could not be removed during grain processing [19].

Scientific studies have shown that the rice rhizosphere secretes specific substances in response to mercury stress [20]. Because rice requires prolonged flooding for normal growth, these conditions enhance the richness and activity of anaerobic microorganisms in rice fields. These rhizosphere microbial communities play a crucial role in converting inorganic mercury (IHg) into methylmercury (MeHg) [21,22]. When rice was cultivated under flooded conditions for 30 days, mercury methylation in the soil reached a relatively stable state [23]. The MeHg content in rice paddies was higher than in dry soils left fallow for a year on the same land, suggesting that anaerobic microorganisms, seasonal flooding, and the presence of iron ions promote the conversion of elemental mercury and IHg into MeHg [21]. Currently, IHg is widely used to evaluate mercury tolerance in rice, while studies directly employing organic mercury as a reagent are limited. Given the prevalence of conditions with elevated organic mercury levels, it is essential to investigate the role of *merA* and *merB* genes in transgenic rice lines to understand their effectiveness in reducing grain mercury accumulation under high organic mercury stress. In this study, we generated transgene lines transformed with *merB* and/or *merA* genes. The influences of the transgenes on plant vegetative growth and some reproduction-related traits were investigated with or without organic mercury stress. At the same time, the grain mercury accumulation was also measured and some useful germplasms were identified for potential application in rice breeding for low-mercury varieties.

## 2. Results

### 2.1. Generation of merA and merB Gene Sequences and DNA Constructs for Rice Transformation

Because there are no homologs of *merA* and *merB* genes in the rice genome, we generated the rice *merA* and *merB* gene sequences based on the published protein sequences encoded by bacterial genes. The encoding sequences were optimized according to rice codon bias to enhance their expression in rice and subsequently synthesized by General Biosystems (Anhui) Co., Ltd. (Chuzhou, China) (Table S1).

**Figure 1:**
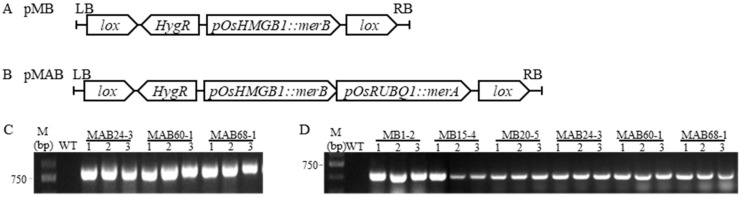
PCR identification of transgenic lines. The recombinant T-DNA in (**A**) pMB and (**B**) pMAB plasmids; (**C**) *merA* gene amplification in three pMAB-transformed lines; (**D**) *merB* gene amplification in three pMB- and three pMAB-transformed lines. Three individual plants from each line were chosen for PCR amplification in C and D. M, molecular weight marker; WT, wild type, Zhonghua 11. The *lox* sites adjacent to both LB and RB provide target sequences for possible transgene excision when necessary.

Two promoters, pOsHMGB1 and pOsRUBQ1, which can drive the gene expression in roots, leaves, flowers, and seeds, were inserted upstream of the synthesized *merB* and *merA* genes, respectively. The constructs were integrated into the pZH109 plasmid, resulting in two recombinant plasmids pMB (containing *merB*) and pMAB (containing both *merA* and *merB*) (Figure 1A,B). Both pMB and pMAB plasmids harbor a *HygR* gene at their LB termini, providing hygromycin resistance.

### 2.2. Molecular Identification of Transgenic Rice Lines

Plasmids pMB and pMAB were transformed into agrobacteria and incubated with seed-derived calli from the japonica rice variety, Zhonghua 11. After screening on the hygromycin-containing MS medium, the resistant calli were transferred to the regeneration medium to produce seedlings, which were subsequently transplanted into soil after a rooting step. A total of 177 T0 transgenic plants were generated, including 95 pMB-transformed plants (referred to as MB hereafter) and 82 pMAB-transformed plants (referred to as MAB hereafter). Within their T2 populations, seven homozygous MB and nine MAB homozygous lines were identified through PCR analysis. Figure 1C,D show representative plants with *merA*-specific amplification in all MAB lines and *merB*-specific amplification in all six lines. No amplification was detected in wild-type (WT) Zhonghua 11 plants.

**Figure 2:**
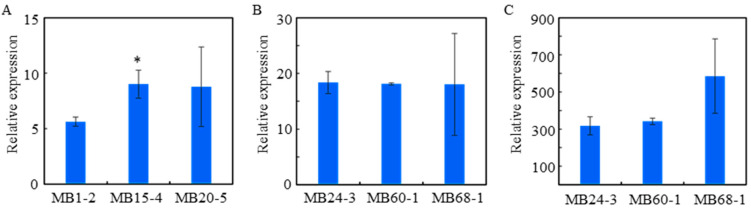
The expression of *merA* and *merB* genes in transgenic lines. (**A**,**B**) Expressions of *merB* gene in MB and MAB lines; (**C**) expression of *merA* in MAB lines, normalized to histone *H3* expression. Asterisk indicates significant differences according to Student’s *t*-test, compared with the lowest expression line. * *p* < 0.05.

At the seedling stage, total RNA was extracted from the leaves of selected transgenic plants, and cDNA was reverse-transcribed using oligo-d(T) primers for RT-qPCR analysis. Six homozygous lines were chosen to test transgene expression, namely MB1-2, MB15-4, MB20-5, MAB24-3, MAB60-1, and MAB68-1. Since neither *merA* nor *merB* are present in the wild-type rice genome, the line with the lowest expression among the three was used as the control for comparison when necessary. Both *merA* and *merB* gene expressions were detected in the transgenic lines, after normalizing to histone *H3* gene expression (Figure 2). Expression variation was observed among the MB lines, but not within those MAB lines. Interestingly, *merB* expression was found to be enhanced above 100% on average when co-expressed with the *merA* gene, compared to the lines expressing *merB* alone (Figure 2A,B). We speculated that this enhancement may be due to the activation of the cis-acting elements in pOsRUBQ1 or *merA*, or possibly due to the overexpression feedback of the *merA* gene, leading to the binding of unknown transcription factors to the pOsHMGB1 promoter, in turn thereby enhancing the expression of the *merB* gene.

### 2.3. Identification of Mercury Resistance of Transgenic and Wild-Type Lines

**Figure 3:**
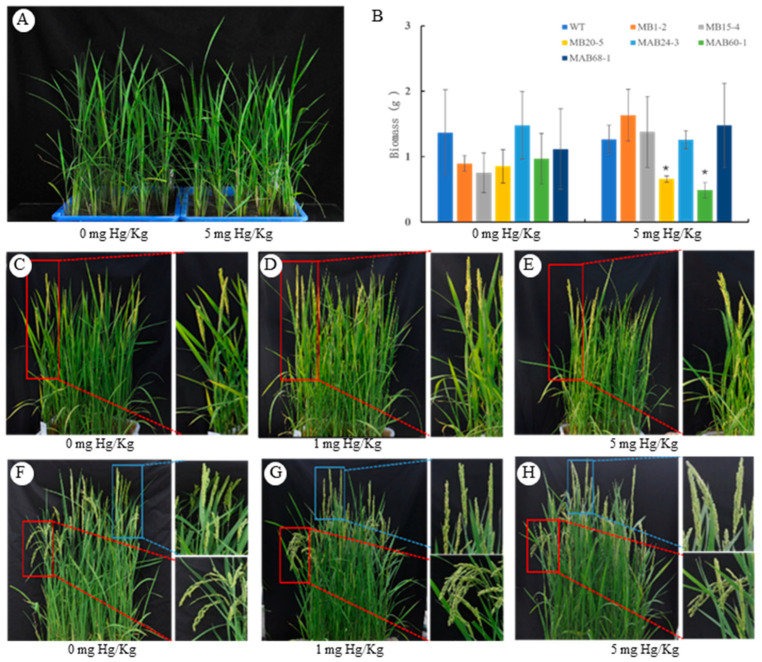
Effect of transgenes on rice vegetative growth (**A**,**B**) and reproduction-related traits (**C**–**H**). (**A**) Pot-grown rice at vegetative growth; (**B**) biomass comparison of dry weight; (**C**–**E**) heading. Panicles in red rectangles shown the heading in wild-type plants; (**F**–**H**) filling. Bowed panicles shown in red rectangles, with upright panicles shown in blue rectangles. Planting was arranged in the pot with 7 rows and in the order (from left to right) of WT, MB1-2, MB15-4, MB20-5, MAB24-3, MAB60-1, Mab68-1, with 5 plants for each genotype. Data in (**B**) shown as mean ± SD from three independent experiments. Asterisks indicate significant differences compared with WT according to Student’s *t*-test, * *p* < 0.05.

To compare the growth between WT and transgenic lines, a pot culture experiment was conducted. Seedlings were planted in plastic pots with seven rows, following the order (from left to right, Figure 3) of WT, MB1-2, MB15-4, MB20-5, MAB24-3, MAB60-1, Mab68-1, with five plants for each genotype (Figure 3). Organic mercury PMA at concentrations of 0, 1, and/or 5 mg/Kg Hg in dry soil was applied to simulate organic mercury stress during rice growth.

#### 2.3.1. The Influence of Transgenes on Biomass Accumulation at Vegetative Growth Stage

About 35 days after transplanting the seedlings into the soil with water, all seedlings from both transgenic and wild-type lines were collected, dried, and weighted. No significant difference in biomass was observed between the wild-type and the transgenic lines, when grown under normal conditions (without mercury), indicating that the transgenes did not affect the biomass accumulation. However, when exposed to 5 mg Hg/Kg mercury stress, the biomasses of the MB20-5 and MAB60-1 lines were significantly reduced by nearly 50%, while another four transgenic lines appeared unaffected (Figure 3A,B). The biomass reduction in the MB20-5 and MAB60-1 lines highlights the growth disadvantages of transgenic lines under stress conditions.

#### 2.3.2. The Influence of Transgenes on Heading and Seeding Filling at Reproductive Growth Stage

About 40 days after the WT seedlings were transplanted into plastic pots, the rice started to undergo heading, indicating that they had entered the reproductive growing stage. But the heading date occurred range from 45 to 48 days after transplantation for most transgenic lines regardless of the presence or absence of mercury stress. This result indicated that then transgene delayed heading for about one week (Figure 3C–E). Seeds’ filling initiated immediately after pollination, until the panicles bowed with adequately filled seeds. Due to the delayed heading in transgenic lines, we observed that most of their panicles remained upright one week after WT heading (Figure 3F,G). This delay in heading and seed filling appeared to be independent of mercury stress or the applied mercury concentration. The observed differences in panicle posture likely resulted from the delayed heading in the transgenic lines, rather than any alteration in the seed-filling process itself.

### 2.4. Yield-Related Traits Under Field Conditions

To further assess growth difference, a field test without mercury stress was conducted in March, 2022, alongside the above pot culture experiment. The plants were harvested in June, and several agronomic traits related to yield were analyzed. As shown in Table 1, only the plant height of transgenic line MB15-4 was significantly higher than that of the WT, with an increase of 15.4%. No significant differences in plant height were observed for the other transgenic lines compared to that of the WT. All transgenic lines exhibited 16–40% fewer productive tilers than the WT. Among the transgenic lines, only MAB60-1 and MAB68-1 showed significantly fewer spikelets per panicle, with reductions of 23.4% and 43%, respectively. The seed-setting ratio and yield per plant of MB15-4 were 37.9% and 41% lower than those of the WT, respectively. In contrast, the 1000-grain weight was notably higher in MB1-2 and MAB68-1, with increases of 17.1% and 20.1% in MB1-2 and MAB68-1, respectively, compared to in the WT. Collectively, each transgenic line exhibited reductions in one to three traits, and seldom increases. MB15-4 exhibited reductions in three yield-related traits, followed by MAB 60-1 and MAB68-1, in which two traits reduced. These results suggested that different transgenic lines were affected to varying degrees, but the most crucial yield traits, yield per plant and 1000-grain weight, showed the minimal impact overall.

### 2.5. Determination of Mercury Content in Rice Grains

The procedure for determining mercury speciation in plants typically involves several steps: sample collection, preparation, mercury extraction, high-performance liquid chromatography (HPLC), and inductively coupled plasma mass spectrometry (ICP-MS). Mercury sample preparation is commonly carried out using one of these three methods: hydrochloric acid digestion (HCl) digestion, Trypsin digestion, and L-cysteine preparation.

**Figure 4:**
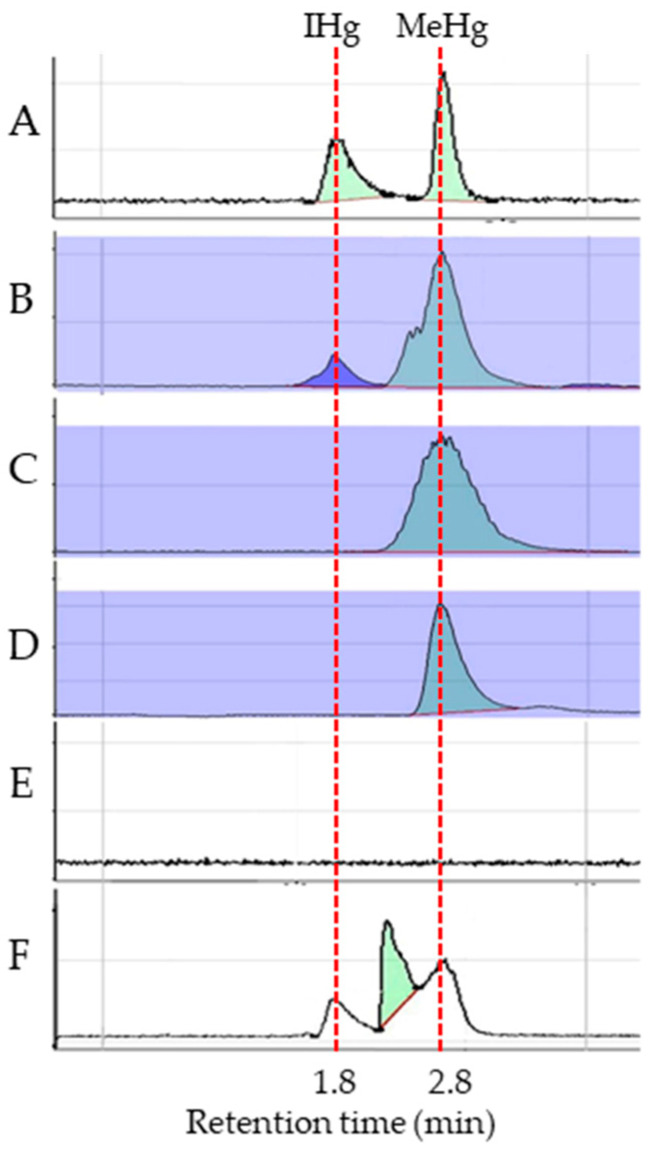
Mercury content determination with different methods in grains. (**A**) Peaks showed IHg (IHg, retention at 1.8 min) and MeHg concentration (MeHg, retention at 2.8 min) of standard samples; (**B**) HCl digestion; (**C**) Trypsin digestion; (**D**) L-Cysteine preparation; (**E**) mock. Sample prepared as sample A without adding grain powder; (**F**) 5 μM PMA spike-in in sample A.

To optimize the mercury sample preparation method in rice, grains from plants grown under 5 mg Hg/Kg mercury treatment were used. As expected, the standard samples of IHg and MeHg showed distinct peaks with ~1 min difference in retention time (Figure 4A). No peaks were detected in the mock sample (Figure 4E). Although MeHg was detectable in samples from all three tested methods, the content of MeHg measured by HCl digestion was ~1.2 times higher than that of the Trypsin digestion or L-cysteine method (Table 2; Figure 4B–D). In addition, only the HCl digestion method successfully detected IHg in rice grains (Table 2 and Figure 4B). A PMA spike-in analysis revealed that PMA was not detectable in harvested grains (Figure 4), indicating that PMA per se does not accumulate in rice grains (Figure 4F). Based on these results, the HCl digestion method was selected for subsequent sample preparation.

**Figure 5:**
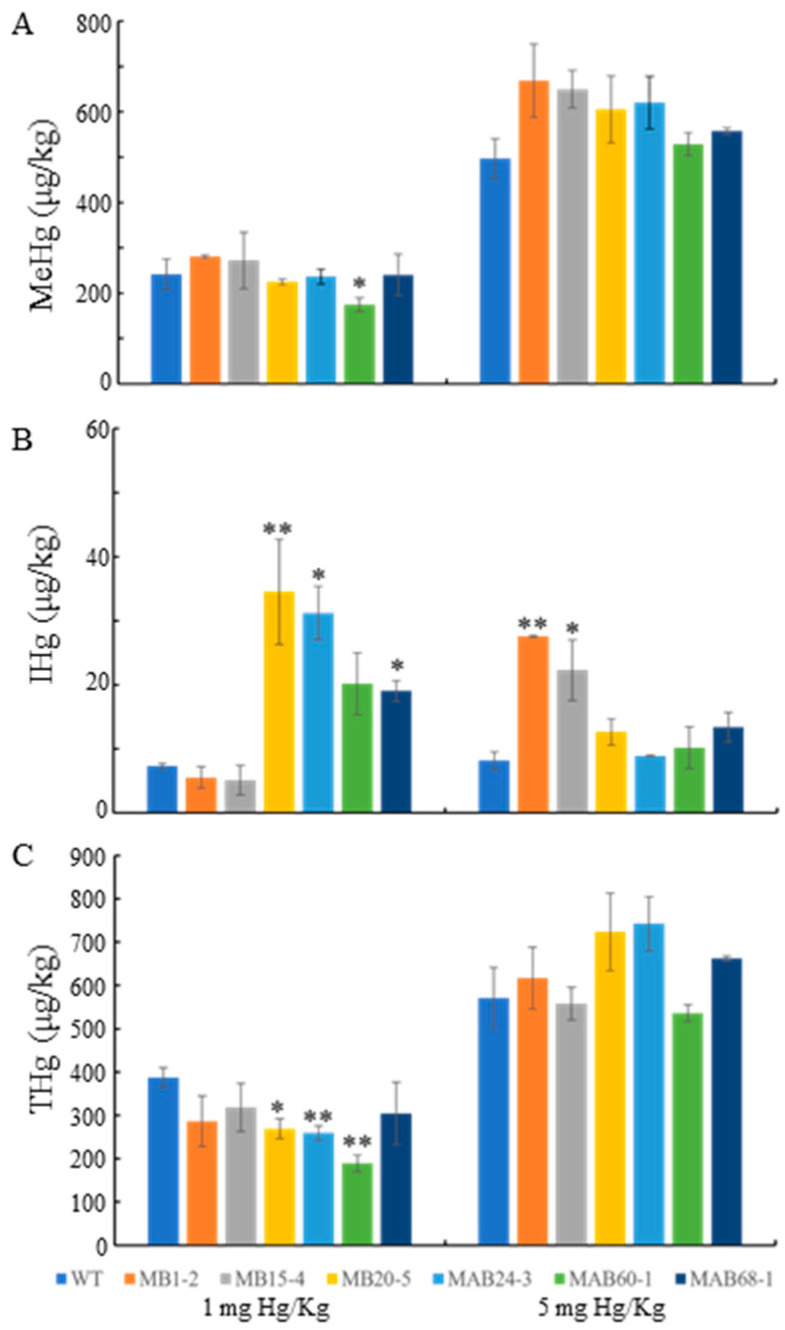
Mercury contents in rice grains. (**A)** MeHg; (**B**) IHg; (**C**) total mercury (THg). Data shown as mean ± SD of three independent experiments. Asterisks indicate significant differences according to Student’s *t*-test, compared with WT. * *p* < 0.05, ** *p* < 0.01.

Next, grain samples, harvested from plants treated with 1 or 5 mg Hg/kg mercury, were prepared using the HCl digestion method and analyzed for mercury speciation using HPLC followingly coupled with ICP-MS. Three genotypes of each MB and MAB line were selected for analysis. Grain MeHg analysis revealed that only MAB60-1 accumulated 72.3% of the MeHg compared to the WT under the 1 mg Hg/kg mercury treatment. However, this reduction was not observed under the 5 mg Hg/kg mercury treatment (Figure 5A). Across all tested samples, 5 mg Hg/kg mercury treatment resulted in 3–5 times higher MeHg accumulation in grains than that of the 1 mg Hg/kg treatment condition. Since MerB catalyzes the breakdown of MeHg into IHg, and MerA converts IHg to elemental mercury, it is reasonable to assume that the MerB activity in the transgenic lines becomes saturated under the 5 mg Hg/kg mercury treatment. Alternatively, the breakdown of the MeHg breakdown process might be inhibited by the accumulation of IHg in MAB lines due to a product-inhibition mechanism.

To explore this possibility, the IHg content was also examined (Figure 5B). Under the 1 mg Hg/kg mercury treatment, two of the three MB lines exhibited no increased IHg accumulation compared to that of the wild type for unknown reasons, while MB15-4 showed the highest IHg levels. Among the MAB lines, MAB24-3 and MAB 68-1 showed significant IHg accumulation in grain, but MAB60-1 did not exhibit significant IHg accumulation. Nevertheless, MAB60-1 has not accumulated significantly high IHg (it still has slightly higher IHg levels than those of the WT). These results suggest that IHg accumulation may inhibit MerB activity. Under the 5 mg Hg/kg mercury treatment, significant IHg accumulation was observed only in two MB lines. Notably, the average IHg content across all test lines was much lower than MeHg levels, accounting for just 8% (1 mg Hg/kg Hg treatment) and 3% (5 mg Hg/kg Hg treatment) of MeHg content, respectively. In summary, MeHg is the predominant mercury speciation in grains, while the low toxicity of IHg makes its contribution almost negligible.

During the grain mercury speciation analysis, only IHg and MeHg were found (Figure 4), allowing us to estimate the total mercury (THg) content in the grains. Similarly to MeHg, the THg content was significantly higher under the 5 mg Hg/kg mercury treatment compared to under the the 1 mg Hg/kg treatment in all lines, indicating a positive correlation between THg accumulation and mercury treatment. Under the 1 mg Hg/kg mercury treatment, the THg content in MB20-5, MAB24-3, and MAB60-1 was significantly lower than in the wild type, with reductions of 30.6%, 33.1%, and 51.1%, respectively.

Based on these results, we conclude that MAB60-1 can effectively reduce both grain MeHg and THg accumulation, followed by MB20-5 and MAB24-3. These three lines show promise as candidate germplasms for breeding low-mercury rice varieties in the future.

## 3. Discussion

Rice is one of the primary food crops in many countries worldwide. With growing global population, the demand for rice cultivation has steadily increased. For instance, China’s rice planting area has remained at approximately 30 million hectares every year for the past 10 years, as announced by the National Bureau of Statistics of China in December 2024. Consequently, the soil–rice system has become the largest constructed wetland ecosystem globally [21]. This cultivation pattern makes rice a critical link between the atmospheric and soil mercury cycles [24].

Mercury is a naturally occurring element widely used in industrial and agricultural processes, ranking third on the substance priority lists. Deposited mercury in soil can be methylated by microorganisms into methylmercury (MeHg), a highly toxic compound even at low concentrations. MeHg is primarily ingested through the food chain, such as fish and rice, with about 95% absorbed via the gastrointestinal tract. It can also enter the body through skin contact and then travel via the bloodstream to the brain and other organs [25]. As rice has become another important source of diet Hg in some areas, developing rice lines with low mercury accumulation can significantly reduce health risks in polluted areas and promote soil-to-atmospheric mercury conversion via transgenic rice, thus regulating the mercury cycle in soil–rice wetland systems.

In this study, organic mercury PMA was selected to simulate mercury stress environment. As PMA was not detected in rice grains under mercury stress (Figure 4), it is speculated that PMA is converted to MeHg by rice rhizosphere microorganisms upon entering soil MeHg and is then transported to rice tissues, accumulating partly in the grains. In addition, physical methods such as alternating wet–dry irrigation or using mineral adsorbents like montmorillonite and medical stones can reduce grain mercury content [26,27]. However, these methods may alter soil properties and cause secondary pollution.

By introducing the *merA* and *merB* genes into rice, MeHg absorbed from the soil can be reduced to IHg, which can then be converted into elemental mercury for volatilization into the atmosphere. Although mercury is naturally volatile, high-temperature cooking cannot fully remove it from rice grains [28]. Hence, developing low-mercury rice lines is essential for ensuring food security. In this study, seven MB and nine MAB homozygous lines were identified, and transgenic lines with high transgene expression (MB lines: MB1-2, MB15-4, and MB20-5; MAB strains: MAB24-3, MAB60-1, and MAB68-1) were selected for mercury content analysis. The MB20-5, MAB24-3, and MAB60-1 lines exhibited significantly reduced total mercury accumulation in grains, with MAB60-1 shown to only have about half the total mercury of that of the wild type.

Hydroponic experiments using PMA and methylmercury chloride (MeHgCl) showed that both compounds induced similar toxic symptoms in rice seedlings. However, MeHgCl displayed greater toxicity, with symptoms appearing more rapidly. Due to environmental and safety considerations, PMA was chosen as the organic mercury reagent for this study.

In this study, stress concentrations of 1 mg Hg/Kg and 5 mg Hg/Kg PMA were selected to assess mercury accumulation for two primary reasons. First, the global average soil mercury content is approximately 1.1 mg/kg soil [4]. Second, prior studies on IHg stress have often used 5 mg Hg/kg soil or higher to simulate mercury stress [29]. It was unclear whether these PMA concentrations would be too severe when we started this study. Our findings revealed that under the 1 mg Hg/Kg PMA treatment, transgenic lines exhibited reduced grain MeHg and THg accumulation compared to those of the wild type. However, even this lower concentration of organic mercury may represent excessive stress, potentially obscuring the full potential of the transgenic lines. Future experiments using lower concentrations of PMA might yield more significant results. On the other hand, the 5 mg Hg/kg PMA treatment led to proportionally higher mercury accumulation in all plants compared to the 1 mg Hg/kg treatment, without negatively impacting plant growth or yield. This finding underscores the capacity of rice to accumulate high levels of mercury in its grains without yield penalties. Given this, breeding rice varieties with low grain mercury accumulation should be prioritized to protect human health.

Li et al. (2020) [30] also reported that transgenic rice with *merA* and *merB* genes, cultivated in soil with 1.6 mg Hg/kg (HgCl2), showed mercury tolerance and reduced grain THg accumulation by 71.6% compared to the wild type. In agreement with the above result, the transgenic MAB60-1 line in this study demonstrated a 51.1% reduction in THg under the 1 mg Hg/kg mercury treatment. These findings highlight a promising strategy for developing low-mercury rice varieties by either de novo co-transformation of *merA* and *merB* into elite rice varieties or crossing these genes into those varieties using pollens from the developed transgenic lines in a cost- and time-saving way.

## 4. Materials and Methods

### 4.1. Plant Materials

The plant materials used in this study included wild-type rice Zhonghua 11 (*Oryza sativa* subsp. Japonica cv.) and two types of transgenic rice lines, MB and MAB lines, obtained from Zhonghua 11 as donor materials for gene transformation. MB rice lines have integrated the *merB* gene in its genome, and MAB rice lines transformed with the *merB* and *merA* genes after an agrobacterial method were used for gene transformation.

### 4.2. Plasmid Construction

The pZH109 plasmid contains the reporter gene *eGFP* and a *HygR* gene at its LB termini for hygromycin resistance. According to the *merA* and *merB* genes of *Pseudomonas adaceae*, based on the codon bias of rice, the *merA* and *merB* gene sequences suitable for expression in rice were optimized [31,32] and synthesized artificially by General Biosystems (Anhui) Co., Ltd. (Chuzhou, China) (Table S1). The above two genes were cloned into plasmid pZH109 to obtain two recombinant plasmids, with one containing only the *merB* gene and the other containing both *merA* and *merB* genes. Then, these plasmids were transformed into agrobacterium for rice transformation.

### 4.3. Pot Culture of Rice

Nine plastic cultivation pots with a length, width, and height of 53 × 34 × 10 cm were prepared. Soil with a dry weight of 7 kg was placed in each pot. The soil was taken from the rice field without mercury pollution and the soil pH was about 6.4. Two concentrations of organic mercury PMA were set in the experiment, 1 mg Hg/Kg and 5 mg Hg/Kg of dry-weight soil. There were three pots for each treatment, resulting in a total of nine pots. After adding the corresponding amount of PMA solution into the pots, 20 L of water was mixed in carefully, and they were left to settle for ~2 weeks under natural conditions for later use. Subsequently, transgenic plant and wild-type seedlings with similar growth status were transplanted into pots, respectively, for growth. Each pot contained the wild type, three MB lines, and three MAB lines. Five plants per line were planted, giving rise to a total of thirty-five plants in each pot.

### 4.4. Molecular Identification of Transgenic Lines

DNA from rice seedlings was extracted by the CTAB method. According to the recombinant plasmid sequence, PCR primers for *merA* and *merB* genes were designed, and the above DNAs were used as the template for PCR amplification to identify those transgenes. Leaf mRNA was extracted according to a RNA extraction kit (TaKaRa Mini BEST Plant RNA Extraction Kit), and mRNA was first used as the template to obtain cDNA by reverse transcription (TaKaRa PrimeScript™ RT reagent Kit, with gDNA Eraser (Perfect Real Time)), and then cDNA was used as the template for quantitative detection and analysis by fluorescent quantitative PCR.

### 4.5. Mercury Speciation Determination

Grain mercury contents were determined using transgenic lines and wild-type seeds, grown under different stress conditions. Genotypes, ZH11, MB1-2, MB15-4, MB20-5, MAB24-3, MAB60-1, and MAB68-1 were used. Combing the mercury concentration of stress and genotypes, a total of 42 seed samples were obtained for further mercury assay.

For the grain sample preparation, the dehusked rice grains were ground to fine powder. HCL digestion referred to the methods by Chen et al. (2020) [33] and GB 5009.268-2016. Then, about 0.1 g of the powder was weighed and put into a 15 mL centrifuge tube, followed by adding 8 mL of 5 mol/L HCl solution and being mixed well. The extraction process was carried out in an ultrasonic water bath at room temperature and kept in darkness for 60 min, with a vigorous vortex every 10 min, 30 s each. The solution was centrifuged at 4 °C × 8000 rpm for 15 min and 2 mL of supernatant was transferred into a 2.0 mL tube and centrifuged again for another 15 min. In total, 0.8 mL of the supernatant was carefully pipetted and put into a new 2.0 mL centrifuge tube, with the drop-by-drop addition of ammonia solution until the pH reached about 5.0. Finally, 0.04 mL L-cysteine solution was added and water was added to adjust the total volume to 2 mL. Then, the mercury extract was obtained after passing the solution through a 0.45 μm filter, and the resulting solution was subject to mercury analysis using HPLC combined with ICP-MS. The Trypsin digestion method [28] and L-cysteine preparation method [34] were carried out using a similar pipeline.

## Figures and Tables

**Table 1:** Yield-related traits of transgenic plants under field condition.

Agronomic Traits	WT	MB1-2	MB15-4	MB20-5	MAB24-3	MAB60-1	MAB68-1
Plant height (cm)	66.7 ± 2.5	71.2 ± 3.7	77.0 ± 1.3 **	68.3 ± 1.1	69.8 ± 2.2	65.6 ± 1.7	65.9 ± 4.5
Productive tiler number	13.8 ± 1.1	11.6 ± 0.5 **	8.4 ± 0.5 **	9.4 ± 1.7 **	8.2 ± 0.8 **	10.3 ± 1.3 **	8.2 ± 1.5 **
Spikelet number per panicle	1480 ± 47.4	1128 ± 339.9	1555 ± 258.3	1032 ± 296.2	1133 ± 305.5	1133 ± 156.9 *	843.3 ± 211.5 **
Seed-setting ratio (%)	47.2 ± 4.9	56.2 ± 5.4	29.3 ± 8.1 *	53.8 ± 4.4	45.5 ± 0.7	46.9 ± 1.7	45.7 ± 3.6
Yield per plant (g)	13.9 ± 2.6	14.5 ± 2.8	8.2 ± 1.4 *	10.6 ± 2.8	12.0 ± 1.5	10.8 ± 1.9	9.5 ± 3.9
1000-grain weight (g)	19.9 ± 1.9	23.3 ± 0.9 *	18.5 ± 1.1	19.3 ± 0.6	23.9 ± 3.4	20.4 ± 0.4	23.9 ± 4.6 *

Data shown as mean ± SD of three independent experiments. Asterisk indicates significant differences (highlighted with bold font) according to Student’s *t*-test, compared with the wild type. * *p* < 0.05, ** *p* < 0.01.

**Table 2:** Mercury determination methods.

Preparation Method	IHg (μg/L)	MeHg (μg/L)
HCl digestion	0.5 × 10^4^	1.929 × 10^4^
Trypsin digestion	0	1.692 × 10^4^
L-Cys preparation	0	1.630 × 10^4^

## Data Availability

Due to privacy issues, the data presented in this study are available on request from the corresponding author.

## Supplementary Materials

The following supporting information can be downloaded at https://www.mdpi.com/article/10.3390/plants14010066/s1, Table S1: Optimized *merA* and *merB* sequences for rice expression.
