# Supplementary material for: Evaluating the Accumulation of Grain Mercury in Engineered Rice Lines Containing merA and merB Genes Under an Organic Mercury-Enriched Condition
Source: Plants (Basel). 2024 Dec 28;14(1):66. doi: 10.3390/plants14010066 (PMC11722697; doi:10.3390/plants14010066)
Supplement: Supplementary file 1 [file plants-14-00066-s001.zip › plants-3362015-supplementary.pdf]

Table S1 Optimized *merA* and *merB* sequences for rice expression.

Gene	Sequences
merA	atgacccaccttaagatcaccggtatgacctgcgactcctgcgctgctcacgttaaggaggctcttgagaaggttcctggtgttcagt<br>ccgctatcgtttcctacgctaagggtgctgctcagcttgctcttgaccctggtaccgctcctgacgctcttaccgctgctgttgctggtct<br>tggttacaaggctatgcttgctgacgctcctcctaccgacaaccgtaccggtcttttcgacaaggttcgtggttggatgggtgctgctg<br>acaagggttccggtggtgagcgtcctcttcaggttgctgttatcggttccggtggtgctgctatggctgctgctcttaaggctgttgag<br>cagggtgctcaggttacccttatcgagcgtggtaccatcggtggtacctgcgttaacgttggttgcgttccttccaagatcatgatccg<br>tgctgctcacatcgctcaccttcgtcgtgagtcccctttcgacggtggtatgcctcctacccctcctaccatccttcgtgagcgtcttcttg<br>ctcagcagcaggctcgtgttgaggagcttcgtcacgctaagtacgagggtatccttgacggtaactccgctatcaccgttcttcacgg<br>tgaggctcgtttcaaggacgaccagtcccttatcgtttcccttaacgagggtggtgagcgtgttgttatgttcgaccgttgccttgttgc<br>taccggtgcttcccctgctatgcctcctatccctggtcttaaggagtccccttactggacctccaccgaggctcttgtttccgacaccatc<br>cctgagcgtcttgctgttatcggttcctccgttgttgctcttgagcttgctcaggctttcgctcgtcttggttcccaggttaccatccttgct<br>cgtaacaccctttttcttccgtgacgacccttccatcggtgaggctgttaccgctgctttccgtgctgagggtatcaaggttcttgagcac<br>acccaggcttcccaggttgctcacgttaacggtgagttcgttcttaccaccggtcacggtgaggttcgtgctgacaagcttcttgttgc<br>taccggtcgtacccctaacacccgttcccttgctcttgacgctgctggtgttaccgttaacgctcagggtgctatcgttatcgacaagg<br>gtatgcgtacctccacccctcacatctacgctgctggtgactgcaccgaccagcctcagttcgtttacgttgctgctgctgctggtaccc<br>gtgctgctatcaacatgaccggtggtgacgctgctatcaaccttaccgctatgcctgctgttgttttcaccgaccctcaggttgctaccg<br>ttggttactccgaggctgaggctcaccacgacggtatcgagaccgactcccgtacccttacccttgacaacgttcctcgtgctcttgct<br>aacttcgacacccgtggtttcatcaagcttgttatcgaggagggttccggtcgtcttatcggtgttcaggttgttgctcctgaggctggt<br>gagcttatccagaccgctgttcttgctatccgtaaccgtatgaccgttcaggagcttgctgaccagcttttcccttaccttaccatggttg<br>agggtcttaagcttgctgctcagaccttcaccaaggacgttaagcagctttcctgctgcgctggttaa
merB	atgaagcttgctccttacatccttgagcgtcttacctccgttaaccgtaccaacggtaccgctgaccttcttgttcctcttcttcgtgagctt<br>gctaagggtcgtcctgtttcccgtaccacccttgctggtatccttgactggcctgctgagcgtgttgctgctgttcttgagcaggctacc<br>tccaccgagtacgacaaggacggtaacatcatcggttacggtcttacccttcgtgagacctcctacgttttcgagatcgacgaccgtc<br>gtctttacgcttggtgccttgacacccttatcttccctgctcttatcggtcgtaccgctcgtgtttcctcccactgcgctgctaccggtgctc<br>ctgtttcccttaccgtttccccttccgagatccaggctgttgagcctgctggtatggctgtttcccttgttcttcctcaggaggctgctgac<br>gttcgtcagtccttctgctgccacgttcacttcttcgcttccgttcctaccgctgaggactgggcttccaagcaccagggtcttgagggt<br>cttgctatcgtttccgttcacgaggctttcggtcttggtcaggagttcaaccgtcaccttcttcagaccatgtcctcccgtaccccttaa
